# Breast Cancer Metastases Presenting as Musculoskeletal Complaints

**DOI:** 10.7759/cureus.113298

**Published:** 2026-07-24

**Authors:** Eric Chun-Pu Chu, Lucina Ng

**Affiliations:** 1 Chiropractic and Physiotherapy Centre, New York Medical Group, Hong Kong, CHN

**Keywords:** bone metastases, breast cancer, breast cancer survivors, chiropractic, chiropractor, musculoskeletal pain, sciatica

## Abstract

Breast cancer often metastasizes to the bone, leading to musculoskeletal symptoms that may mimic benign conditions. Patients with undiagnosed or recurrent malignancies may initially present to first-contact providers, including chiropractors, with non-specific spinal or musculoskeletal pain. Here, we extend the series with two additional cases, highlighting risks of delayed diagnosis, the role of imaging, and chiropractic care. In Case 1, a 62-year-old woman with a history of breast carcinoma presented with progressive left-sided sciatica-like symptoms (pain radiating from the sacroiliac region to the lower extremity), revealing progressive metastases on MRI (e.g., L5 vertebral collapse with retropulsion and severe stenosis) and PET/CT (e.g., new intraspinal extensions at C2, T2, and T4). Multidisciplinary intervention, including radiotherapy, targeted therapy (letrozole, palbociclib, denosumab), and conservative chiropractic management, reduced Visual Analog Scale (VAS) pain scores from 7/10 to 3/10 and improved quality of life from 58/100 to 86/100. In Case 2, a 44-year-old woman presented with constant lumbar pain (7/10 intensity), leading to the discovery of multilevel vertebral collapses (e.g., C7, T3, T5, T8, and L2) and abnormal marrow signals on MRI, confirmed as invasive ductal carcinoma via biopsy and PET/CT. These cases underscore the need for vigilance in patients with an oncologic history presenting with musculoskeletal pain. These clinical outcomes highlight the synergy of an interdisciplinary management model, where primary oncology therapies (radiotherapy and systemic targeted agents) successfully achieve metabolic and structural stabilization of the metastatic lesions, while concurrent, low-force supportive chiropractic care safely focuses on mechanical symptom mitigation and preserving the patient's quality of life.

## Introduction

Metastatic breast cancer continues to pose significant clinical challenges despite advances in targeted therapies and supportive care, which have extended survival and transformed the disease into a chronic, relapsing condition for many patients [1]. With increased survivorship comes greater complexity in care, as patients must manage not only ongoing oncologic treatment but also the long-term effects of metastasis, therapy-related side effects, and comorbidities that collectively impact daily function and quality of life [1].

Cancer recurrence does not always present with classic oncologic warning signs [2]. Instead, relapse may manifest subtly or atypically, often mimicking common musculoskeletal complaints such as back pain or sciatica, especially in patients who consider their cancer to be well controlled [3]. This diagnostic challenge is particularly pronounced in individuals with a history of bone metastases, who are at increased risk for skeletal complications yet may attribute new symptoms to benign musculoskeletal causes or routine physical activity.

Given that the incidence of breast cancer is projected to increase in regions like Hong Kong, non-oncology primary care providers, including chiropractors, must maintain a heightened index of suspicion when encountering new-onset musculoskeletal complaints in this demographic [4]. Epidemiological screening data demonstrate that a history of cancer is more predictive of current spinal malignancy in patients presenting with low back pain than any other patient-reported clinical sign, carrying a substantial positive likelihood ratio (+LR) of 7.3 [5]. Clinicians are positioned to detect early warning signs of disease recurrence, especially when symptoms are disproportionate to typical musculoskeletal findings or are accompanied by neurological changes [5]. However, there is limited literature on the role of chiropractors in the detection and management of metastatic disease, and guidance for safe, integrative care in this population remains sparse [6, 7]. We previously reported three cases of breast cancer presenting as chiropractic complaints [4-6]. This case report describes two patients: a 62-year-old female breast cancer survivor who presented with presumed sciatica and a 44-year-old female with constant lumbar pain and no known prior oncologic history, both of whom were found to have metastatic spinal disease. These cases illustrate the pivotal role of musculoskeletal clinicians in unmasking cancer recurrence, the complexity of survivorship, and the need for interdisciplinary collaboration to optimize patient outcomes.

## Case presentation

Case 1

A 62-year-old female presented to a chiropractic clinic with a six-month history of progressive left-sided sciatica-like symptoms, which began following a hiking trip. The patient described the onset of pain localized to the left sacroiliac region, radiating to the buttock and left lower extremity, accompanied by tension, tingling, and a “cotton-like” sensation in the left foot. She also reported intermittent tingling along the left lateral thigh and occasional tenderness in the right lateral leg. Her symptoms gradually worsened, significantly affecting her mobility and daily activities. She had attempted self-management with rest and over-the-counter analgesics but noted minimal relief.

**Figure 1:**
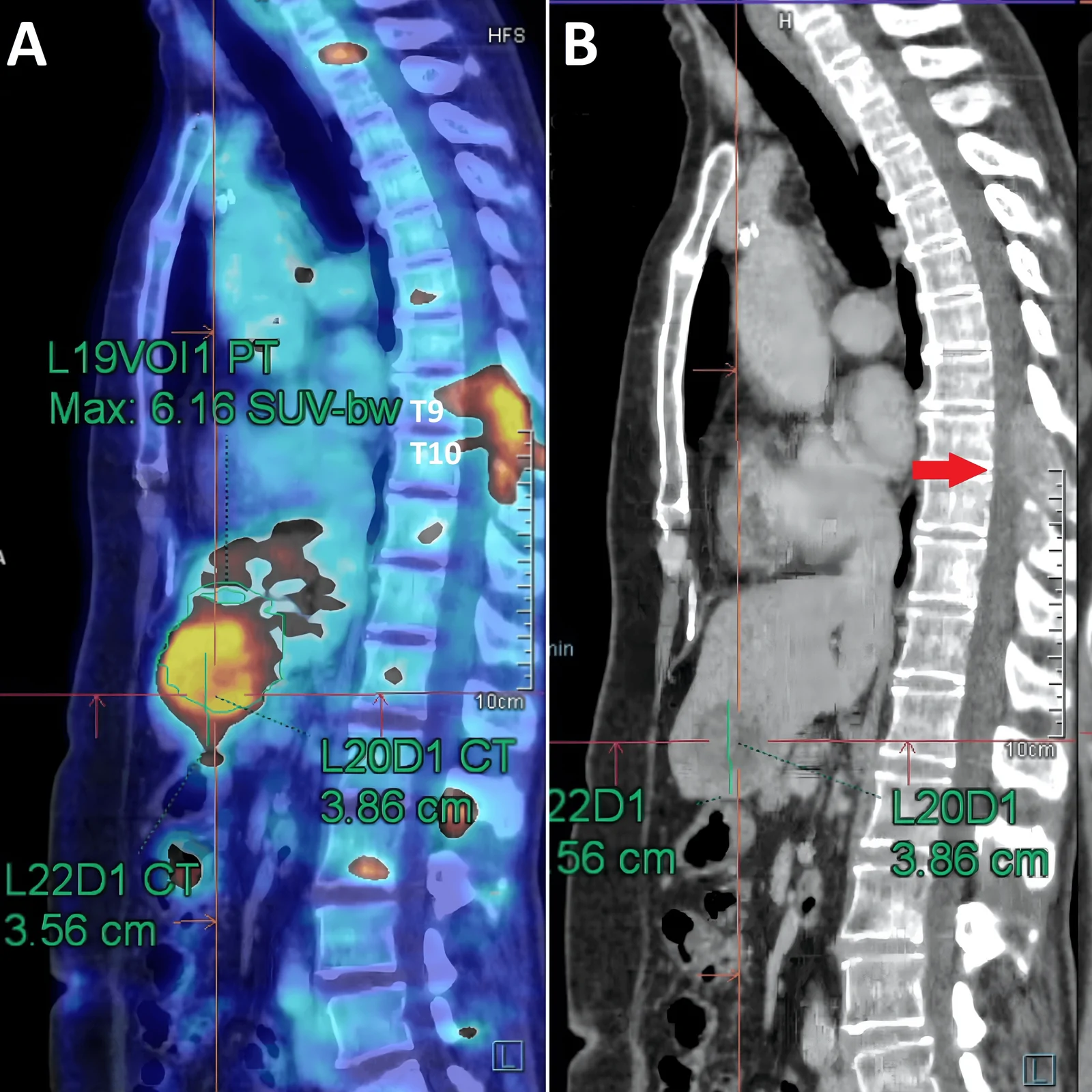
PET/CT images demonstrating left breast carcinoma with multiple bony metastases. (A) Coronal fused PET-CT image shows multiple hypermetabolic osseous lesions, including prominent metastases involving the posterior element of T9 and the spinous process of T10 with extension into the spinal canal (color overlay). (B) The corresponding coronal CT image highlights the destructive metastatic lesion at T10 (red arrow), with associated spinal canal narrowing and cord compression.

Her medical history was notable for left breast carcinoma diagnosed 26 months prior, initially detected by a PET scan revealing invasive carcinoma with metastases to regional lymph nodes, liver, and multiple bones (Figure 1). The patient received targeted therapy, including letrozole, denosumab, and palliative radiotherapy to the T9-T10 region. A follow-up PET scan after treatment showed a positive response with reduced metabolic activity in the breast, lymph nodes, liver, and bone metastases and resolution of spinal cord compression; however, new osseous lesions were identified at the L5 superior endplate and T8 vertebra. Believing her cancer was stable, the patient resumed her usual activities, including hiking, and reported no significant symptoms until the onset of her current complaints.

On chiropractic examination, the patient exhibited mild forward head posture and a left pelvic tilt. Lumbar spine range of motion was restricted: flexion limited to 40° (normal 60°), extension to 15° (normal 25°), and left lateral flexion to 20° (normal 25°), each movement eliciting left buttock pain. Palpation revealed tenderness and hypertonicity of the left sacroiliac joint, piriformis, and gluteus medius muscles, with segmental restriction at L4-L5 and S1-S2. The straight leg raise (SLR) test was positive at 30° on the left, reproducing tingling in the knee, while the right SLR was negative. Neurological examination identified diminished light touch sensation along the left L5 dermatome, 4/5 strength in the left hip abductors and ankle dorsiflexors, and preserved deep tendon reflexes bilaterally.

**Figure 2:**
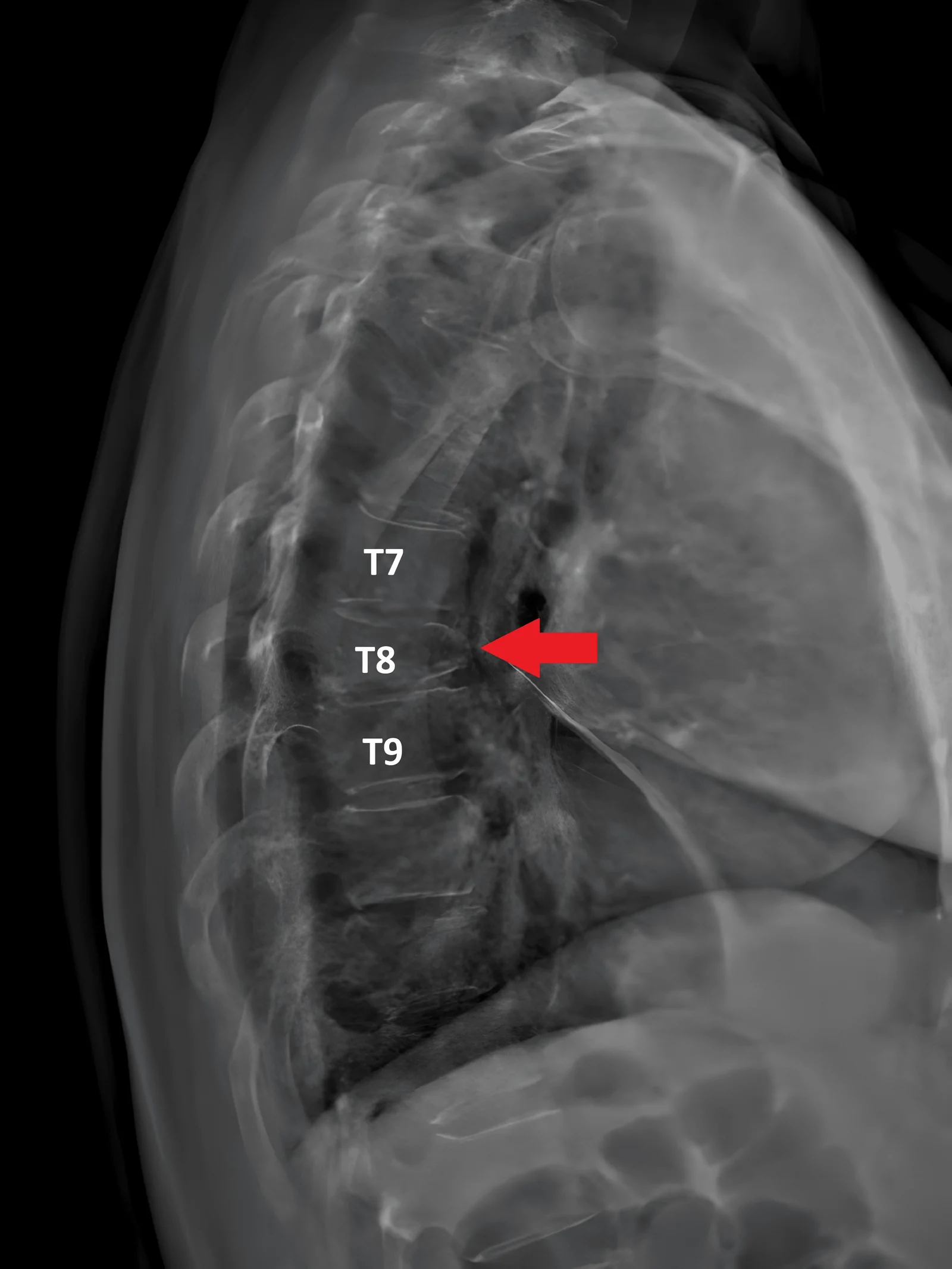
Radiograph of the thoracic spine. Osteopenia with mild osteoporotic fractures is identified at the mid thoracic spine (red arrow).

**Figure 3:**
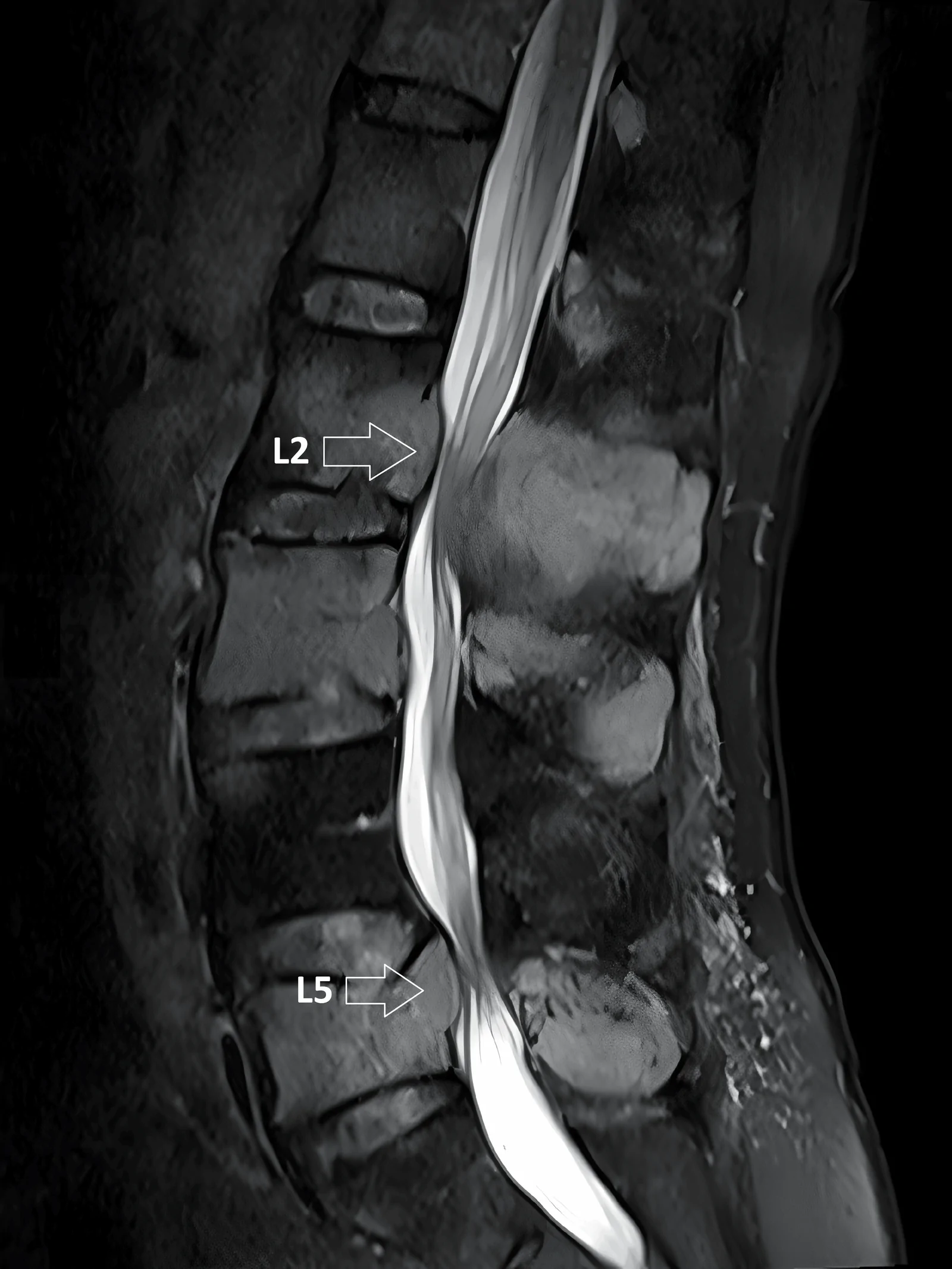
MRI of the lumbar spine. At L2 and L5 levels, severe stenosis is seen in relation to the retropulsion and expansion effect of the bone metastasis (white arrows).

Given the patient’s oncologic history and the severity of symptoms, the chiropractor ordered a full-spine X-ray, which showed osteopenia and a mild osteoporotic fracture in the mid-thoracic spine (Figure 2). Due to a mismatch between the imaging findings and the clinical presentation, an urgent MRI was obtained. The MRI revealed multiple bone metastases (Figure 3), including partial collapse of the L5 vertebral body with retropulsion into the spinal canal and severe stenosis at this level. Recognizing the risk of neurological deterioration from bone metastasis, the chiropractor referred the patient to the radiology and oncology teams working at the same medical center for further evaluation.

**Figure 4:**
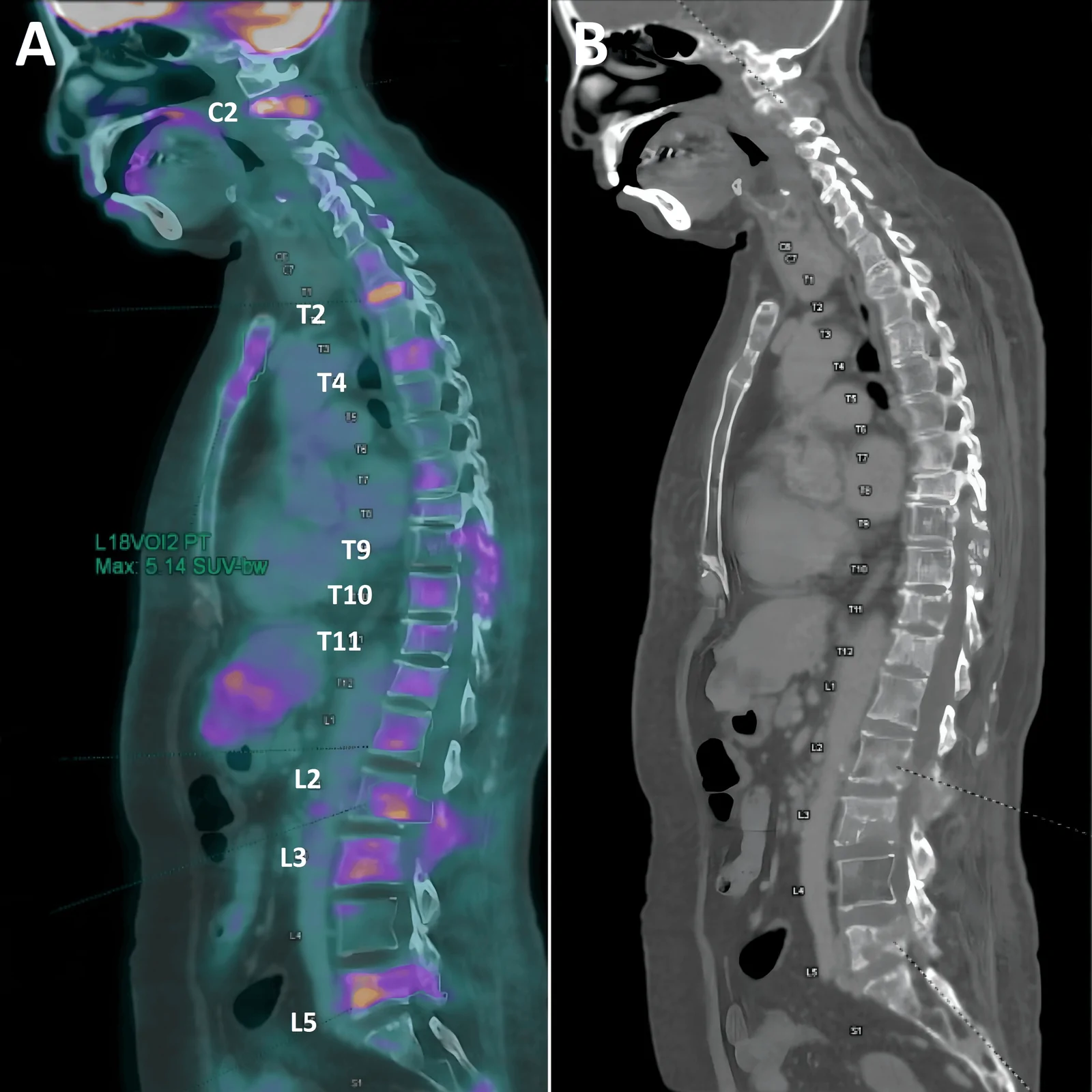
PET-CT images demonstrating multiple new and progressive skeletal metastases 26 months after initial diagnosis A) Coronal fused PET-CT image shows widespread hypermetabolic osseous lesions involving the vertebral column (C2, T2, T4, T9–T11, L2, L3, L5), pelvis, sternum, bilateral scapulae, bilateral ribs, both humeri, and left femur. Note intraspinal extension at multiple levels (e.g., C2, T9–T11). (B) The corresponding coronal CT image better delineates the osseous destructive changes and anatomical extent of the metastases.

A PET scan performed by the radiology team confirmed disease progression, with multiple new and worsening avid lesions in the left breast, nodal regions from the neck to the pelvis, liver, and skeleton. Notably, new intraspinal tumor extensions were identified at C2, T2, and T4, raising concerns for cord compression (Figure 4). The patient was promptly treated with 10 sessions of radiotherapy targeting the C2, T2, T4, and L5 regions, along with resumption of letrozole and the addition of palbociclib for three months. Post-treatment PET imaging demonstrated metabolic resolution of the segment I hepatic lesion, partial improvement in the segment III hepatic lesion, and stabilization of skeletal metastases, though some intraspinal extension persisted.

The patient was promptly treated with 10 sessions of radiotherapy targeting the C2, T2, T4, and L5 regions, along with resumption of letrozole and the addition of palbociclib for three months. Post-treatment PET imaging demonstrated metabolic resolution of the segment I hepatic lesion, partial improvement in the segment III hepatic lesion, and stabilization of skeletal metastases, though some intraspinal extension persisted. Following stabilization, the patient was referred back to the chiropractor for supportive care. A tailored three-month management plan was instituted, comprising low-force chiropractic adjustments (activator method) focused on the L4-L5, S1-S2, and mid-thoracic segments, gentle soft tissue therapy of the piriformis and gluteal muscles, and individualized rehabilitation exercises (daily lumbar stabilization and a progressive walking program). Given the profound structural vulnerabilities present-including L5 vertebral collapse, retropulsion, severe central canal stenosis, and active cervical and thoracic metastatic lesions-strict clinical safety protocols were enforced. Traditional high-velocity, high-force spinal manipulation was avoided. The schedule included three visits per week for the first month, followed by weekly sessions for the subsequent two months. At three-month reassessment, the patient reported marked improvement in sciatica-like symptoms (VAS score reduced from 7/10 to 3/10), increased lumbar range of motion, and decreased tingling, with persistent but stable left L5 sensory deficit. No adverse events occurred during chiropractic care, and the patient continued to receive regular oncologic follow-up.

Case 2

A 44-year-old female software developer presented to the chiropractic clinic complaining of low back pain that began one week ago. She described the pain as a constant, dull ache localized in the lumbar region, with an intensity of 7 out of 10 on the pain scale. Her work involves prolonged periods of sitting, and she reported that the pain was exacerbated by prolonged sitting and standing, but there was no radiating pain down the legs or any associated numbness or tingling sensations. She denied any recent injuries or specific events that could have triggered the onset of the pain. She did not use tobacco and drinks alcohol socially, averaging two to three drinks per week. She denied any recreational drug use. Her exercise routine was minimal, though she expressed a desire to start weight control, and she lost three pounds in one month. The patient's symptoms interfered with her daily activities and overall quality of life.

The patient's past medical history was unremarkable. She underwent an appendectomy at the age of 22 and a cesarean section at age 30. She reported no chronic illnesses and has no known drug allergies. Currently, she occasionally uses ibuprofen to manage her pain, taking it as needed. Family history revealed hypertension in her mother and diabetes mellitus in her father, who passed away from a myocardial infarction at the age of 68. She had no siblings with significant health issues.

During the physical examination, the patient demonstrated a limited range of motion in the cervical spine, particularly noticeable during right rotation and lateral flexion to the right side. Upon further evaluation, palpation of the cervical region revealed muscle tightness and mild tenderness along the right paraspinal muscles of the neck. The lumbar spine was also assessed, correlating with her chief complaint of low back pain; palpation elicited tenderness in the lower lumbar paraspinal muscles and severe pain in lumbar flexion. Orthopedic testing, including the straight leg raise test, was negative for radicular pain, and neurological examination showed no deficits in motor or sensory function in the lower extremities. Reflex assessment was normal and symmetrical in both upper and lower limbs. The patient's posture was observed, displaying a slight anterior pelvic tilt and a forward head posture, which are common postural deviations that may contribute to spinal discomfort. Despite having no prior known history of primary oncologic disease, several high-risk 'red flags' were identified during the initial intake. The patient reported a 3-lb unexplained weight loss over the preceding month, persistent night pain that prevented sleep, and localized bony tenderness that was vastly disproportionate to a standard mechanical strain. She was sent for imaging using the EOS® X-ray imaging system (EOS imaging, Paris, France) for posture and spinal integrity assessment.

**Figure 5:**
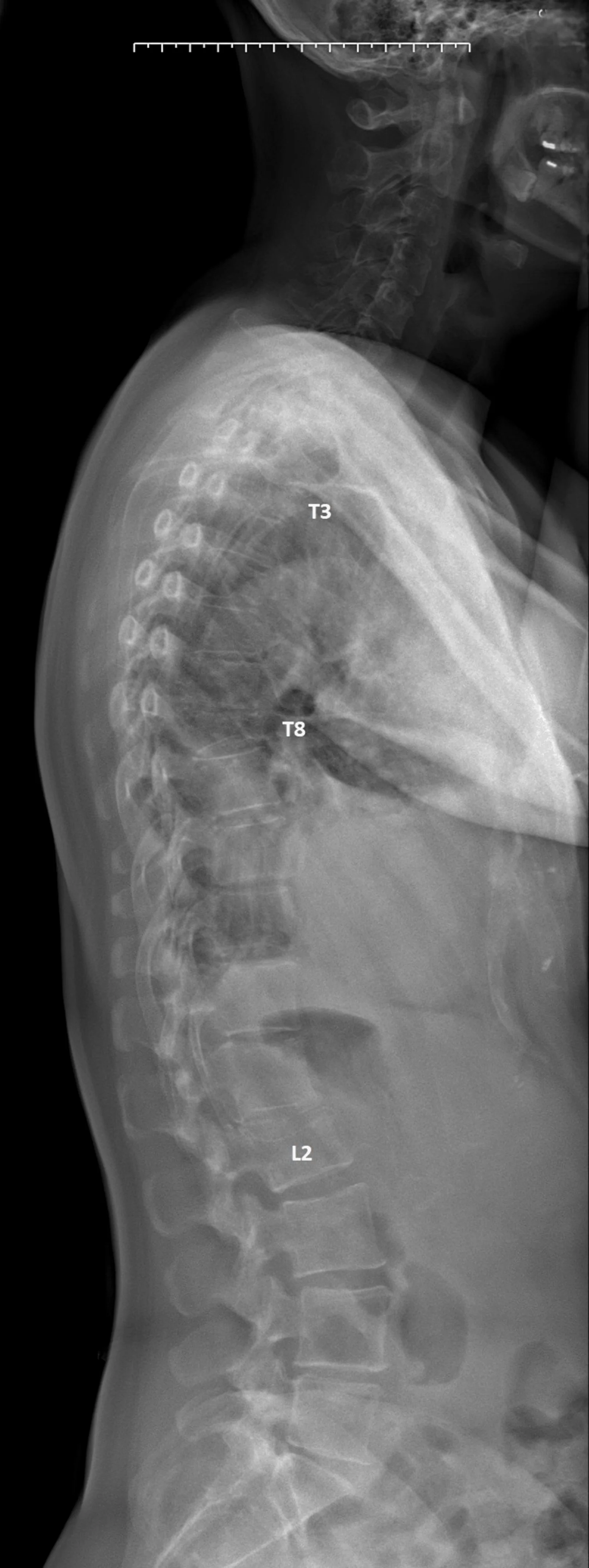
Sagittal thoracic and lumbar plain radiographs demonstrating multi-level structural compromise Significant loss of anterior and posterior vertebral body height is noted at the T3, T8, and L2 levels, representing pathological compression fractures secondary to osteolytic metastatic infiltration.

The next day, upon evaluation of the EOS® X-ray, the imaging revealed a forward head posture and an anterior pelvic tilt, indicating postural imbalances that could be contributing to the patient’s symptoms. More critical, however, were the findings within the thoracic spine. The X-ray showed a notable reduction in vertebral height at the T3, T8, and L2 levels (Figure 5). These changes suggested the presence of compressive changes possibly due to degenerative conditions or previous trauma. Such vertebral alterations could be impacting the structural integrity of the spine and potentially influencing nerve function and muscle tension in the area, correlating with the patient’s reported symptoms and physical examination findings. The chiropractor sent her for an MRI evaluation the next day to rule out spinal stenosis and other pathologies, prompted by red flags including unremitting pain despite ibuprofen use, recent unexplained weight loss (three pounds in one month), and severe pain on lumbar flexion without a clear mechanical cause.

**Figure 6:**
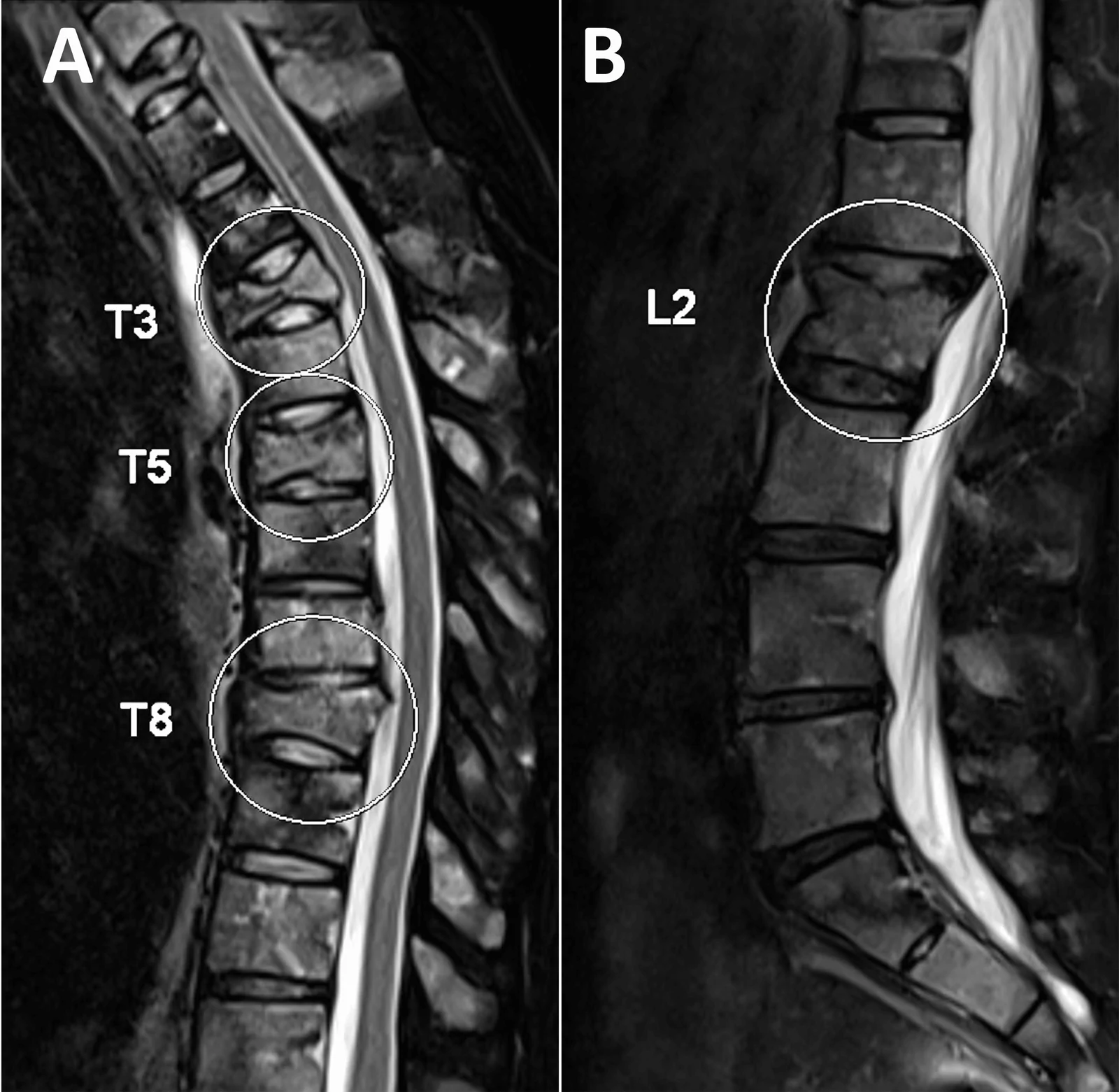
MRI of the thoracic and lumbar spine. (A) MRI T1 image of the thoracic spine identified partial collapse at T3, T5 and T8. Retropulsion of vertebrae is causing mild central canal stenosis at T3 and T8 level. Diffuse hypointense and hyperintense metastatic marrow infiltrations are visible throughout the column. (B) MRI T1 image of the lumbar spine identified partial collapse of L2 vertebra with minimal retropulsion. L1-2 disc herniation is causing moderate stenosis of the central canal and left subarticular zone, driving the patient's constant mechanical back pain and elevated risk of neurological deficit. Abnormal marrow signal is seen involving the visible skeleton, suspicious for bone metastases.

**Figure 7:**
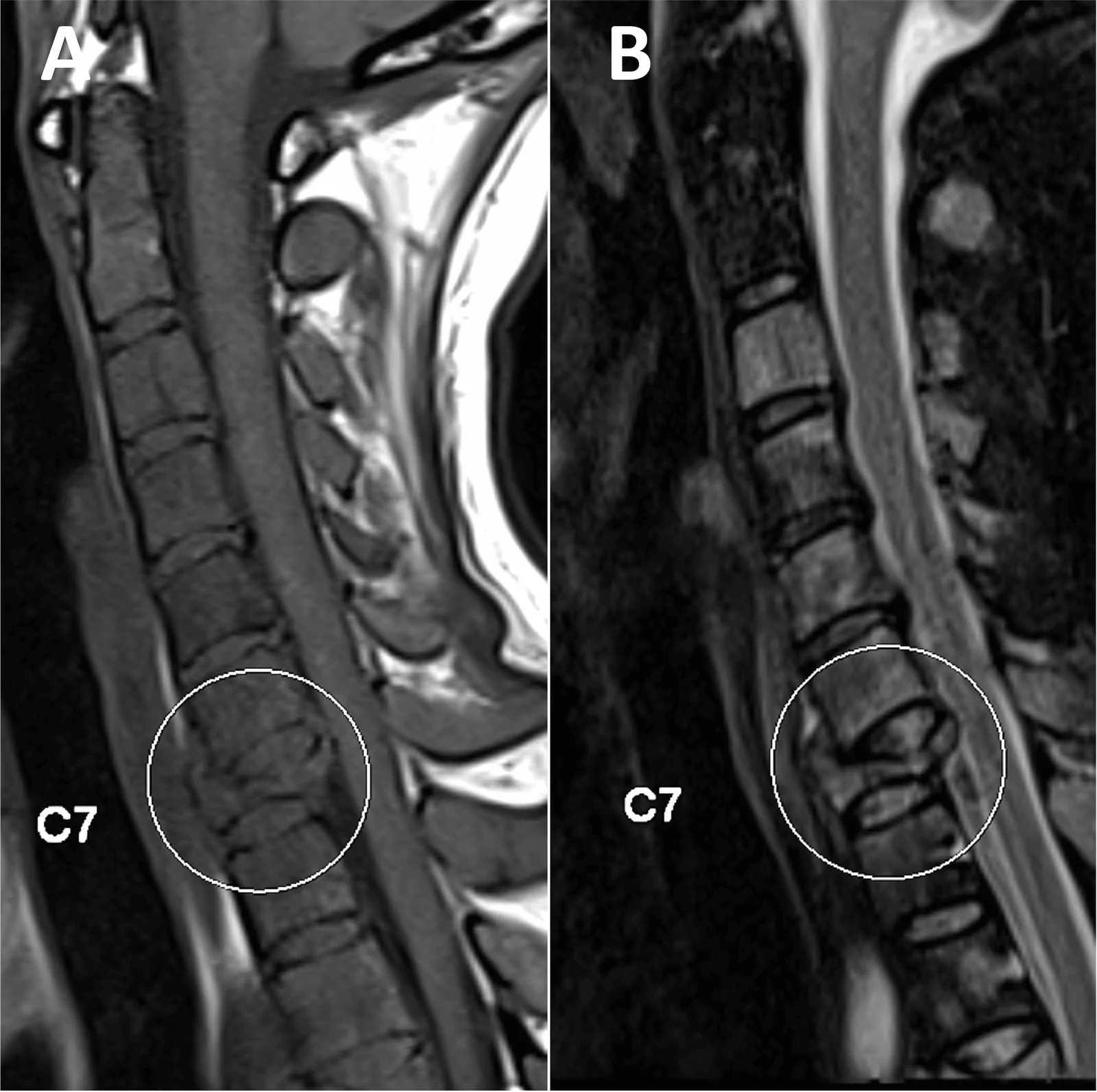
MRI of the cervical spine. (A) T2-weighted image shows the collapse of the C7 vertebra with moderate retropulsion. Moderate stenosis of the central canal is noted. (B) T1-weighted shows abnormal marrow signal involving C2 to C7. Findings are suspicious for a marrow infiltrative process; in particular, bone metastasis merits consideration.

On the third day, her MRI of the spine revealed partial collapse of the C7, T3, T5, T8, and L2 vertebrae, with retropulsion at C7, T3, T8, and L2 contributing to central canal stenosis at these levels (Figure 6). Abnormal marrow signals were detected from C2 to C7, raising concerns for a marrow-infiltrated process, with bone metastasis being a primary consideration (Figure 7). Abnormal marrow signals were also present throughout the visible skeleton, further supporting suspicions of bone metastases. These comprehensive findings indicate a severe underlying condition likely affecting multiple areas of the spine. As the chiropractor works as the primary healthcare practitioner, she was immediately sent to the local hospital for an oncology examination with PET scans. A biopsy was performed, and she was diagnosed with invasive ductal carcinoma.

Following diagnosis, the patient was managed through oncology with PET scans confirming metastatic spread and biopsy verifying invasive ductal carcinoma. Oncologic treatment included targeted radiotherapy to affected vertebral levels and systemic chemotherapy (e.g., regimens potentially involving agents like letrozole or similar based on tumor profile), aimed at disease stabilization.

Following the definitive diagnosis of advanced systemic recurrence, the patient was immediately transitioned to a coordinated multidisciplinary care framework. Primary oncology interventions included targeted local palliative radiotherapy to ensure structural stability and provide pain relief. This was combined with first-line systemic endocrine therapy consisting of the aromatase inhibitor letrozole. Post stabilization, chiropractic interventions were integrated for supportive care. A short-term, four-week trial of care was initiated, consisting of twice-weekly sessions. This included gentle, low-force cervical and lumbar mobilization (e.g., Cox flexion-distraction to address L1-L2 disc-related stenosis) alongside myofascial release of the cervical paraspinal muscles and mild postural corrective exercises to address her forward head posture. The patient reported initial symptom relief from these conservative measures, with ongoing oncologic follow-up recommended to monitor progression.

Key clinical milestones and oncologic management steps are summarized in Table 1.

**Table 1:** Chronological timeline of clinical milestones, diagnostic intervals, and interventions. VAS: Visual Analog Scale; NRS: Numeric Rating Scale

Clinical Phase	Case 1 (62-year-old female)	Case 2 (44-year-old female)
Symptom Onset	Week 0: Progressive left-sided sciatica-like pain radiating down the lower extremity. Baseline pain: 7/10 VAS.	Week 0: Constant, severe localized lumbar back pain. Unexplained 3-lb weight loss. Baseline pain: 7/10 NRS.
Initial Presentation	Week 2: Patient seeks chiropractic evaluation. Intake reveals historical primary breast carcinoma (2020).	Week 1: Patient seeks chiropractic evaluation. Physical exam reveals antalgic gait and severe multi-segmental tenderness.
Imaging Escalation	Week 2 (Day 2): Emergency lumbar MRI ordered; reveals L5 collapse with retropulsion and canal stenosis.	Week 1 (Day 3): Radiographs and urgent spinal MRI ordered; reveal multi-level collapses (C7, T3, T5, T8, L2).
Definitive Diagnosis	Week 3: PET/CT tracking confirms active metabolic metastases at C2, T2, T4, and L5.	Week 2: Biopsy and advanced metastatic workup confirm occult primary invasive ductal breast carcinoma.
Primary Oncological Management	Weeks 4–6: Fast-tracked referral; local palliative radiotherapy administered (30 Gy/10 fractions) to L5. Initiation of letrozole, palbociclib, and denosumab.	Weeks 3–8: Immediate transition to oncology; emergency stabilization, palliative radiotherapy to high-risk zones, and systemic chemotherapy (docetaxel/cyclophosphamide).
Supportive Care Integration	Weeks 7–19: Post-radiotherapy clearance obtained. Initiation of a three-month trial of gentle instrument-assisted mobilization (Activator) at remote, non-metastatic compensation zones.	Week 12: Integrated into post-stabilization supportive team. Commencement of a four-week trial of twice-weekly gentle, low-force cervical/lumbar adjustments.
Long-Term Follow-up & Outcomes	Month 58: Formal tracking endpoint. Complete metabolic resolution of L5 lesion; stable remaining bones. VAS reduced to 3/10; SF-36 QOL increased from 58 to 86.	Month 12: Standard monitoring checkpoint. Successful systemic disease monitoring, stable weight, and managed NRS pain score of 4/10 under collaborative observation."

Previously published cases (Cases 3-5)

Our prior reports included three cases that complement the current extension by highlighting similar patterns of musculoskeletal presentations in metastatic breast cancer, often with delayed recognition due to benign-appearing initial imaging. Succinctly, Case 3 involved a 36-year-old postpartum woman with persistent neck pain, leading to MRI detection of C3 vertebral plana and metastasis, managed with radiotherapy, chemotherapy, and chiropractic rehabilitation, improving quality of life from 60% to 90% [4]. Case 4 described a 68-year-old woman with chronic low back pain exacerbating to include paresthesia, where initial radiographs suggested hemangioma, but MRI and PET/CT revealed diffuse marrow metastases, prompting urgent oncologic referral [5]. Case 5 featured a 41-year-old woman with recurrent low back pain radiating to the leg, diagnosed via MRI as an L5 metastasis, treated with gentle traction, soft tissue manipulation, radiotherapy, and chemotherapy, resulting in pain reduction from 6/10 to 2/10 and sustained relief over 18 months [6]. The new cases build on our prior publications by emphasizing multilevel spinal involvement (new Case 2) and post stabilization chiropractic supportive care (new Case 1), contrasting with the pregnancy-related diagnostic challenges in prior Case 3 and the long latency (20 years) in prior Case 5.

## Discussion

This case series extension reinforces that breast cancer metastases can masquerade as common musculoskeletal issues, such as sciatica-like symptoms or localized back pain, particularly in patients with a history of stable disease. In Case 1, the patient's symptoms post-hiking trip initially suggested a mechanical etiology, but her oncologic background prompted advanced imaging, uncovering progressive metastases with spinal involvement. Similarly, Case 2 revealed multilevel vertebral collapses mimicking degenerative changes, aligning with our previous cases (Cases 3-5) [4-6], where delays in recognition risked neurological compromise.

These findings compare with broader literature, where musculoskeletal presentations occur in metastatic cancer cases, often involving the axial skeleton and leading to underdiagnosis in primary care [7-9]. Integrative care, as demonstrated here, echoes studies showing chiropractic interventions (e.g., low-force adjustments) can improve pain and QOL in oncology patients, with VAS reductions of 30%-50% when combined with radiotherapy [10]. Key lessons include the utility of MRI and PET/CT for detecting occult progression, as X-rays alone may underestimate severity by missing early marrow changes [11].

Given the multi-modal nature of the management pathways in this series-where patients concurrently received local palliative radiotherapy, bone-targeted agents, and systemic anticancer therapies-the individual therapeutic contribution of supportive manual care cannot be isolated. The observed clinical and functional improvements must be interpreted cautiously as the cumulative outcome of an integrated, multidisciplinary care model. While radiotherapy remains the gold standard for achieving biological local tumor control and reducing bone pain, modified low-force rehabilitative care was integrated strictly to alleviate secondary mechanical strain and improve positional tolerance [12]. Future prospective studies are needed to delineate the specific interaction effects between manual supportive care and medical oncological regimens.

Limitations include the small series size, the retrospective nature potentially introducing recall bias, and selection bias toward cases presenting to chiropractic settings, limiting generalizability. Future studies could explore prospective protocols for screening musculoskeletal complaints in cancer survivors. Based on the diagnostic timelines observed in this case series, it is hypothesized that optimizing pathways for primary musculoskeletal clinicians-including potential direct-access frameworks for advanced diagnostic imaging such as MRI-could facilitate earlier detection of occult spinal metastases [13]. However, translating this clinical observation into systemic healthcare policy requires further investigation. Broad health economics and health service research are needed to formally evaluate the cost-effectiveness, safety, and diagnostic utility of such expanded access models within integrated oncology networks.

## Conclusions

This series highlights the insidious musculoskeletal presentation of breast cancer metastases and the benefits of prompt imaging and collaborative care. Clinicians should suspect metastases in patients with a cancer history and unexplained pain, ordering an MRI for red flags like progression despite conservative treatment. Integrative chiropractic care can be considered as a post-oncologic stabilization part of the oncology rehabilitation team to enhance quality of life.
